# Pharmacokinetic Study of Oral ^14^C-Radiolabeled Hyzetimibe, A New Cholesterol Absorption Inhibitor

**DOI:** 10.3389/fphar.2021.665372

**Published:** 2021-05-28

**Authors:** Jianwei Liao, Xin Wang, Zhenyu Li, Dongsheng Ouyang

**Affiliations:** ^1^ Department of Clinical Pharmacology, Xiangya Hospital, Central South University, Changsha, China; ^2^ Institute of Clinical Pharmacology, Central South University, Changsha, China; ^3^ Zhejiang Hisun Pharmaceutical Co., Ltd, Taizhou, China; ^4^ Department of Geriatric Medicine, Xiangya Hospital, Central South University, Changsha, China; ^5^ Hunan Key Laboratory for Bioanalysis of Complex Matrix Samples, Changsha, China

**Keywords:** radiolabel, pharmacokenitics, phase I, healthy men, hyzetimibe, ezetimibe

## Abstract

**Background and objectives:** Hyzetimibe is a candidate drug being investigated as the second-in-class cholesterol absorption inhibitor; it lowers plasma levels of low-density lipoprotein cholesterol (LDL-C) by blocking the Niemann-Pick C1-like 1 protein, a transporter mainly expressed in the intestine that allows dietary cholesterol to enter the body from the intestinal lumen. Previous studies on the metabolism of hyzetimibe in healthy volunteers were not enough to show the biotransformation and excretion pathway; in particular, whether hyzetimibe maintains pharmacological action for duration sufficient to pass through the hepatic-intestinal circulation remains unknown. Furthermore, it remains unclear whether the differences between the chemical structures of ezetimibe and hyzetimibe would result in different pharmacokinetic characteristics. Given that the molecular target is in the intestine and the substantial hepatic-intestinal circulation is a metabolic characteristic of the drug, a study of hyzetimibe as an oral ^14^C-radiolabeled drug, compared with routinely metabolized drugs, would play an important role in uncovering pharmacokinetic details.

**Methods:** After an overnight fast and before taking medication, six healthy male volunteers swallowed an investigational product suspension containing 20 mg/∼100 μCi of ^14^C-labeled hyzetimibe as a single dose. Whole-blood, plasma, urine, and fecal samples were collected, and hyzetimibe and its metabolites were measured. Pharmacokinetic variables of hyzetimibe and its metabolites were calculated and statistically analyzed according to obtained concentration data. Safety data were collected throughout the study.

**Results:** The major metabolite detected in plasma was hyzetimibe-glucuronide, which accounted for 97.2% of the total plasma radioactivity. The mean cumulative excretion of total radioactivity of the dose was 16.39% in urine and 76.90% in feces. Unchanged drug and hyzetimibe-glucuronide were identified as the major components in the feces and the urine, respectively. The main metabolic conversions of hyzetimibe were glucuronidation (M1), mono-oxidation (M4), and mono-oxidation with additional sulfonation (M7). Hyzetimibe was considered generally safe and well tolerated.

**Conclusion:** This study of ^14^C-radiolabeled hyzetimibe provides a full profile of the biotransformation and excretion routes of hyzetimibe to improve the understanding of the pharmacokinetic characteristics of hyzetimibe. The changed hydroxyl group in the hyzetimibe structure made it easier for that drug, compared with ezetimibe, to combine with glucuronic acid and subsequently increased the urinary excretion of hyzetimibe vs. ezetimibe. These differences highlight the need to investigate in more detail the different pharmacokinetic impacts on the efficacy and safety of hyzetimibe and ezetimibe.

## Introduction

Hyzetimibe (HS-25) is a second-in-class, potent, orally administered lipid-lowering compound that is currently under development. It can selectively inhibit the absorption of cholesterol and related phytosterols in the small intestine. Clinical trials of hyzetimibe have been carried out in America and China since 2012 (NCT03413462, NCT03433196, NCT03464682, NCT02087917), and a new drug application had been submitted to the China Center for Drug Evaluation of National Medical Products Administration. The chemical structure of hyzetimibe is similar to that of ezetimibe, a first-in-class cholesterol absorption inhibitor.

Both drugs can maintain their pharmacological action for a long time through hepatic-intestinal circulation, and the special metabolic route makes them have little clinical interaction with the combined drugs such as Atorvastatin.

The results of preclinical and clinical studies of hyzetimibe have shown that hyzetimibe notably lowers the levels of low-density lipoprotein cholesterol (LDL-C), total cholesterol (TC), non-high-density lipoprotein cholesterol (non-HDL-C), and apolipoprotein B (apo-B) with fast and stable efficacy. Long-term use of hyzetimibe is necessary, so it is particularly important to obtain information about the excretion, mass balance, and metabolism in humans. A previous study in animals and in healthy adult volunteers (Ruan et al., 2014) determined the metabolic characteristics of hyzetimibe in animals according to preclinical pharmacokinetic studies and discovered that hyzetimibe was metabolized to its active metabolite by different isoforms of UDP-glucuronosyltransferase in the intestine and liver; however, the metabolite profiles, excretion pathways, and mass balance of hyzetimibe (the excretion of hyzetimibe and all metabolites in urine and feces) in humans remained unknown. In that study, it was not clear whether the small differences in the chemical structures between ezetimibe and hyzetimibe would result in different pharmacokinetic characteristics. Ruan et al. (2014) also examined the metabolic characteristics of the hepatic-intestinal circulation of hyzetimibe, because its molecular targets were in the intestine. Those authors determined that a study of orally administered ^14^C-radiolabeled hyzetimibe would offer substantial details about the drug metabolism. Therefore, this study was developed to assess the distribution of hyzetimibe and its metabolites in whole blood and plasma; to explore the pharmacokinetics of total radioactive hyzetimibe and hyzetimibe-M1; to examine the mass balance, excretion pathways, and biotransformation pathways; and to report on the safety and tolerability of a single oral dose of ^14^C-labeled hyzetimibe in healthy male volunteers to provide a reference for the rational use of the drug.

## Methods

### Overall Study Design

This was an open-label, single-dose study. The screening/baseline period of the study was approximately seven days, and the observation period was approximately seven-to-ten days, for a total study time of approximately two weeks. Whole-blood, plasma, and pooled urine and fecal samples were obtained on a regular basis to recover the radioactive dose. Safety data were collected throughout the study. The length of the observation period might have been shortened or prolonged if deemed appropriate according to emerging data. After the mass balance cumulative recovery of >85% had been achieved or <1% of dose had been collected in urine and feces within two consecutive days, volunteers were permitted to leave the study center. If emerging data showed that adequate dose recovery was not achieved after ten days (i.e., day 11), those volunteers were asked to continue providing urine and/or fecal samples at twenty-four-hour intervals. When the plasma drug radioactivity concentration was less than two times the plasma background value within two consecutive time points, the collection of radioactive blood samples was stopped. All adverse events (AEs) were collected for each volunteer from the time of informed consent until the follow-up visit or the last day of urine/fecal collection.

### Study Population Selection

Healthy male volunteers between 18 and 45 years of age with a body mass index between 19 and 26 kg/m^2^ were enrolled in the study. Participants with irregular bowel movements or with long-term exposure to radioactivity—from radioactive working conditions, substantial radiation exposure (more than two chest/abdominal computed tomography scans or more than three other X-ray examinations), or those who participated in radiopharmaceutical labeling trial within one year of this study—were excluded from the study. The approval and implementation of this study strictly followed the ethical principles of the Declaration of Helsinki, the International Conference on Harmonization (ICH)/Good Clinical Practice (GCP), and applicable regulatory requirements. The Medical Ethics Committee of the First Affiliated Hospital of Suzhou University approved this study (Approval No. 091-1). All volunteers gave written informed consent and satisfied the study inclusion/exclusion criteria of the clinical study protocol before they were administered the investigational product.

### Doses and Treatment Regimens

Each bottle of hyzetimibe solid powder for oral administration contained 20 mg/∼100 μCi of ^14^C-labeled hyzetimibe. The radiochemical purity was 100.00%, and the specific activity was 5.033 μCi/mg (equivalent to 2.13 mCi/mmoL).

In the phase II clinical study on the lipid-lowering effect and efficacy of hyzetimibe, observation of the 20-mg dose group confirmed good safety and effectiveness. The 20-mg dose was proposed for daily clinical administration and so was used as the oral dose in this study.

On day 1, after an overnight fast for at least 10 h and no water for at least 1 h before medication administration, volunteers swallowed the investigational suspension containing 20 mg/∼100 μCi of ^14^C-labeled hyzetimibe as a single dose. The planned dose of 100 μCi represented a small risk level of human radiation exposure (International Commission on Radiological Protection, 1991; International Commission on Radiological Protection, 2007; Code of Federal Regulations, 2020). According to our preliminary estimation, the effective dose of the labeled drug used in this study would not exceed 0.0297 mSv, which aligns with the radiation dose limit recommended by the International Commission on Radiologic Protection (International Commission on Radiological Protection, 1991; International Commission on Radiological Protection, 2007; Code of Federal Regulations, 2020). After ingestion of the administered dose, the vial used for administration was rinsed multiple times with purified water. Volunteers ingested the investigational suspension, the rinses, and additional purified water for a total fluid ingestion of 240 ml. After the volunteers received the medicine, the residual radioactive dose in the drug container and related articles were determined.

The use of radioactive compounds was in accordance with the requirements of the radiation safety license of the study site (Jiangsu environmental radiation license A0666). The treatment of radioactive waste generated during the study was in accordance with urban radioactive waste management measures and with the basic national standards of ionizing radiation protection and radiation source safety (GB18871-2002) issued by General Administration of Quality Supervision, Inspection and Quarantine of China.

### Sample Collection

To determine the plasma concentrations of hyzetimibe and its metabolites and to measure the total radioactivity, blood samples were collected before the dose and at 0.25, 0.5, 1, 1.5, 2, 2.5, 3, 4, 5, 6, 8, 10, 12, 24, 36, 48, 72, 96,120, 168, and 240 h after the dose. Whole-blood samples were collected before and at 0.5, 1, 2, 6, 12, and 24 h after the dose to obtain the ratio of total radioactivity in whole blood to plasma. Urine samples were collected before and at 0–4, 4–8, 8–12, 12–24, 24–48, 48–72, 72–96, 96–120, 120–144, 144–168, 168–192, 192–216, and 216–240 h after the dose, and the sample weights were recorded. Fecal samples were collected before and at 0–24, 24–48, 48–72, 72–96, 96–120, 120–144, 144–168, 168–192, 192–216, and 216–240 h after the dose, and the sample weights were recorded. To determine metabolic conversion and the metabolic rate, plasma samples were collected before and at 0.25, 1, 2.5, and 8 h after the dose. Four mixed urine samples and four mixed fecal samples at different time periods were also used for metabolic conversion and metabolic rate assessments.

### Sample Preparation and Bioanalysis

Blood samples were vortexed, weighed, and combusted in a Sample Oxidizer 501 (Harvey, United States) to detect total radioactivity. Plasma was well vortexed and weighed to detect total radioactivity. Urine was well mixed and weighed to detect total radioactivity. Pooled fecal homogenates (in a 50% isopropanol solution) were weighed, dried, and combusted in a Sample Oxidizer 501 (Harvey, United States) to detect total radioactivity. Plasma samples were processed by protein precipitation using acetonitrile (1/4, v/v) to determine the plasma concentrations of hyzetimibe and hyzetimibe-M1.

For the pharmacokinetic analysis, total radioactivity levels in whole-blood and plasma samples were quantified using liquid scintillation counting in a Tri-Carb 4910 TR liquid scintillation spectrometer (PerkinElmer, United States). Plasma concentrations of hyzetimibe and its metabolites in the volunteers were determined at different time points using a validated liquid chromatography/tandem mass spectrometry (MS/MS) method (Chen et al., 2015). The plasma samples were analyzed by ultra-performance liquid chromatography (LC-30AD; Shimadzu, Japan combined with mass spectrometry (UPLC-MS/MS). The high performance liquid chromatography (HPLC) column was a Hypersil GOLD C18 system (50 × 2.1 mm, 1.9 μm; kept at 40°C, mobile phase flow rate of 0.4 ml/min; Thermo, United States with Waters Critical Clean PER 735001035 tubing (Waters, United States). The mass spectrometer (QTRAP^®^5500, AB SCIEX, United States) was equipped with a heated electrospray ionization interface. The mobile phase consisted of mobile phase A (water) and mobile phase B (methanol). From 0.00 to 0.80 min, the gradient solvent composition was 95% mobile phase A and 5% mobile phase B. From 0.80 to 2.80 min, mobile phase A gradually decreased to 5%, and mobile phase B gradually increased to 95%. From 2.80 to 3.50 min, the gradient solvent composition was 5% mobile phase A and 95% mobile phase B. From 3.51 to 4.50 min, the gradient solvent composition was 95% mobile phase A and 5% mobile phase B. The data processing systems were Analyst®1.6.3 and MultiQuant™ 3.0.2 (AB SCIEX, United States. The sample sizes were 5 µl each. The linear range of determination was 0.05–20 ng/ml for hyzetimibe and was 1–400 ng/ml for hyzetimibe-M1.

The total radioactivity levels in the homogenates of urine and feces were measured by liquid scintillation counting in a Tri-Carb 4910 TR liquid scintillation spectrometer (PerkinElmer, United States).

Selected plasma, urine, and fecal samples were mixed, extracted and analyzed by reversed-phase HPLC (LC-2030C, Shimadzu, Japan) with online/offline radioactivity detection (vARC Radio-Assay System, Model 3, AIM, United States) for metabolic profiling. The main radioactive metabolites in samples of mixed plasma, urine, and feces were identified by HPLC combined with low-energy radioactivity monitor and mass spectrometry (LC/RAM/MS). The RP-HPLC column was an ACE C18 (ACE, British) (150 × 4.6 mm, 3.0 μm, kept at 40°C, mobile phase flow rate of 0.7 ml/min. The mass spectrometer (Thermo LTQ Orbitrap XL LC-MS/MS System, Thermo Scientific, United States) was equipped with a heated electrospray ionization interface. The sample spray was delivered through a formate-amine solution. The data processing system was Xcalibur (Thermo Scientific, United States).

### Pharmacokinetics and Safety Measurements

Pharmacokinetic outcome measures included the following: maximum plasma concentration (C_max_); time to maximum plasma concentration (T_max_); area under concentration-time curve from zero (predose) extrapolated to infinity (AUC_0-∞_); area under the concentration-time curve from zero (predose) to the time of the last quantifiable concentration (AUC_0-t_); apparent terminal elimination half-life (t_1/2_); and mean residence time (MRT).

To evaluate the safety and tolerability of a single oral dose of hyzetimibe in healthy male volunteers, adverse events (AEs), vital signs, physical examination, 12-lead electrocardiogram (ECG), and laboratory variables (hematology, chemistry, urinalysis, and hemagglutination) were assessed. AEs were recorded from the time of informed consent to the end of the study or to the early withdrawal assessment. AEs were graded using the National Cancer Institute Common Terminology Criteria for Adverse Events grading system, version 4.03.

### Statistical Methods

The sample size of the study was not based on hypothesis testing. Rather, on the basis of similar previously conducted studies of mass balance, a sample size of six volunteers was considered sufficient to gain adequate absorption, distribution, metabolism, and excretion information while exposing as few volunteers as possible to the investigational product and procedures. The pharmacokinetic analysis set included six volunteers who received one dose of hyzetimibe. These six volunteers were included in the safety population. Because this was an exploratory study, the data analysis relied mainly on descriptive statistics and did not involve formal power calculations or model-based statistical analyses. Pharmacokinetic variables, plasma concentrations, urine and fecal samples of ^14^C-labeled radioactivity data, and safety/tolerability were summarized using appropriate descriptive statistics [e.g., mean, (SD), coefficient of variation (CV%), geometric mean, geometric coefficient of variation (GCV%), minimum, median, and maximum]. Figures of the mean concentration-time data were developed to present plasma ^14^C-labeled radioactivity concentrations, plasma hyzetimibe concentrations, and plasma hyzetimibe-M1 concentrations.

## Results

### Demographic Characteristics

The enrolled participants were healthy Chinese men. The mean age of the participants was 30.2 years, and the mean height was 169.25 cm. The mean body mass index was 21.88 kg/m^2^, within the protocol-specified range of 19–26 kg/m^2^.

### Pharmacokinetic Results

**FIGURE 1:**
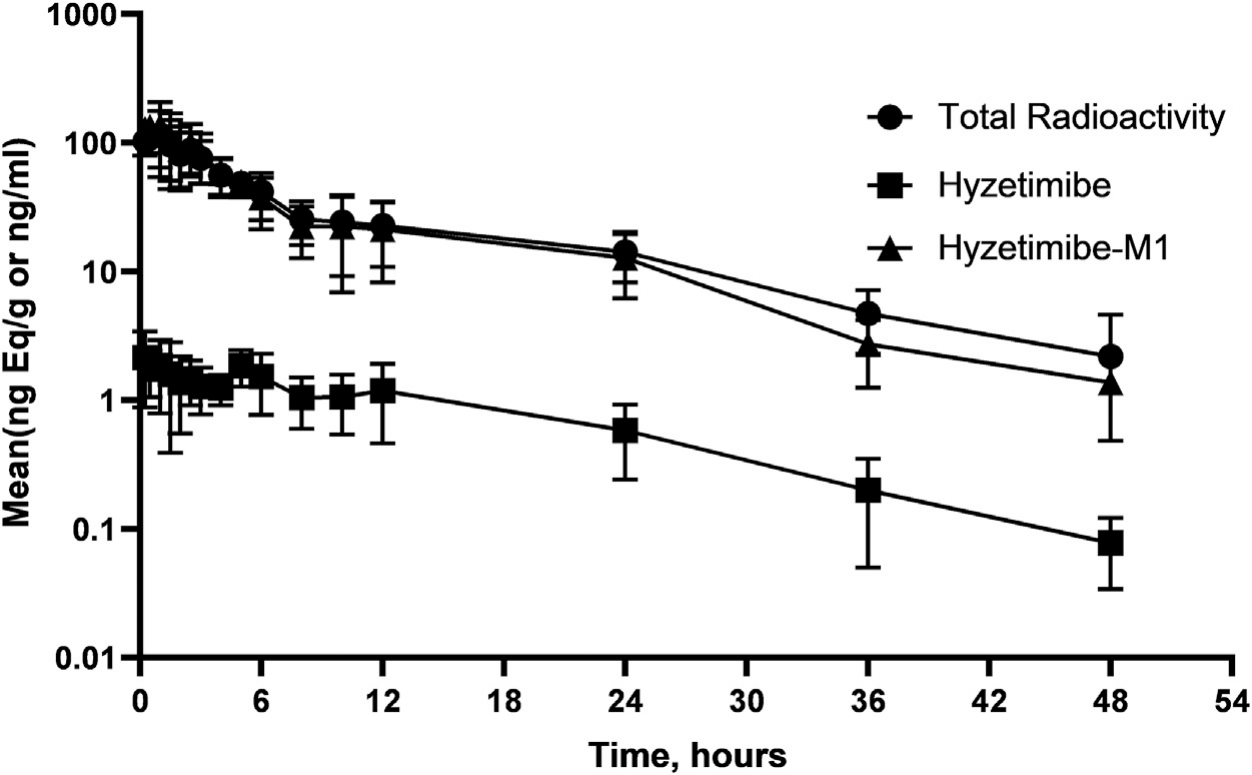
Mean (SD) hyzetimibe and hyzetimibe-M1 concentration-time and total radioactivity-time profiles in plasma.

Mean concentration-time profiles in plasma after a single dose of ^14^C-labeled hyzetimibe are shown in Figure 1. The mean hyzetimibe-M1 concentrations were close to the total radioactivity, but the mean hyzetimibe concentrations were notably lower than the total radioactivity. Pharmacokinetic variables are shown in Table 1. The total radioactivity in plasma was determined by liquid scintillation counter. The median T_max_ of total radioactivity was 1.00 h (minimum, maximum: 0.250, 2.50 h). The mean maximum concentration (C_max_) of total radioactivity was 139 ± 43.8 ng Eq/ml. The mean concentration of radioactivity was 14.2 ± 6.01 ng Eq/ml at 24 h after administration. Only a small amount of radioactivity was detected in the plasma of three volunteers at 48 h after administration. In the other three volunteers, plasma levels were below the lower limit of radioactivity quantification (3.76 ng Eq/g). The mean AUC_0-t_ of the total radioactivity in plasma was 961 ± 307 h × ng Eq/ml. The mean MRT_0-t_ of total radioactivity was 10.5 ± 2.22 h. The mean t_1/2_ of total radioactivity was 13.2 ± 5.81 h.

**TABLE 1:** Summary of pharmacokinetic parameters and total radioactivity of hyzetimibe and hyzetimibe-M1.

	Variable	Unit of measure	Number of screenings						N	Mean (SD)
			007	010	013	015	016	020		
Total radioactivity	C_max_	ng × Eq/mL	129	227	113	113	121	130	6	139 (43.8)
	T_max_	h	0.25	1	2.5	2.5	0.5	1	6	1.00 (0.250, 2.50)^a^
	AUC_0-t_	h × ng Eq/mL	965	1,450	886	1,040	490	935	6	961 (307)
	AUC_0-∞_	h × ng Eq/mL	1,030	1,530	1,050	1,100	621	1,010	6	1,060 (290)
	MRT_0-t_	h	13.1	10.2	12	11.8	7.1	8.98	6	10.5 (2.22)
	t_1/2_	h	10.1	14.1	24.4	8.25	11.3	10.9	6	13.2 (5.81)
^14^C-hyzetimibe	C_max_	ng/mL	4.38	3.76	2.22	2.61	1.67	2.2	6	2.81 (1.04)
	T_max_	h	0.25	1.5	4.95	0.25	4.92	4.88	6	3.19 (0.250, 4.95)^a^
	AUC_0-t_	h × ng/mL	36.3	44.4	27.7	45.1	15.1	28.5	6	32.9 (11.4)
	AUC_0-∞_	h × ng/mL	37.6	46	29.8	45.7	17	29.5	6	34.3 (11.1)
	MRT_0-t_	h	17.3	11.9	13.6	14.2	8.25	13.1	6	13.1 (2.97)
	t_1/2_	h	7.53	9.8	14.8	6.24	6.95	9.5	6	9.13 (3.10)
^14^C-hyzetimibe-M1	C_max_	ng/mL	157	265	138	134	143	157	6	166 (49.6)
	T_max_	h	0.25	1	2.5	2.5	0.5	1	6	1.00 (0.250, 2.50)^a^
	AUC_0-t_	h × ng/mL	907	1,410	834	1,080	486	987	6	950 (303)
	AUC_0-∞_	h × ng/mL	922	1,430	893	1,090	585	1,000	6	987 (277)
	MRT_0-t_	h	11.3	8.6	9.86	11.5	6.3	8.54	6	9.35 (1.97)
	t_1/2_	h	6.43	9.04	15.5	5.57	10.3	7.91	6	9.13 (3.56)

AUC_0-t_, area under the concentration-time curve from zero (predose) to the time of the last quantifiable concentration; AUC_0-∞_, area under concentration-time curve from zero (predose) extrapolated to infinity; C_max_, maximum plasma concentration; MRT_0-t_: mean residence time; SD, standard deviation; T_max_, time to maximum plasma concentration; t_1/2_, apparent terminal elimination half-life.

a Median (range).

A validated LC-MS/MS method was used to determine the plasma concentrations of hyzetimibe and hyzetimibe-M1. The median T_max_ in plasma was 3.19 h (minimum, maximum: 0.250, 4.95 h) for hyzetimibe and was 1.00 h (minimum, maximum: 0.250, 2.50 h) for hyzetimibe-M1. The mean maximum concentration (i.e., C_max_) was 2.81 ± 1.04 ng/ml for hyzetimibe and was 166 ± 49.6 ng/ml for hyzetimibe-M1. One volunteer had a plasma concentration at 48 h after administration that was below the lower limit of quantification (hyzetimibe: 0.0500 ng/ml, hyzetimibe-M1: 1.00 ng/ml). The mean plasma concentration of the other five volunteers was 0.0779 ± 0.0438 ng/ml for hyzetimibe and was 1.37 ± 0.888 ng/ml for hyzetimibe-M1 at 48 h after administration. The mean AUC_0-t_ in the plasma was 32.9 ± 11.4 h × ng/ml for hyzetimibe and was 950 ± 303 h × ng/ml for hyzetimibe-M1. The mean ± SD MRT_0-t_ was 13.1 ± 2.97 h for hyzetimibe and was 9.35 ± 1.97 h for hyzetimibe-M1. The t_1/2_ was 9.13 ± 3.10 h for hyzetimibe and was 9.13 ± 3.56 h for hyzetimibe-M1. The exposure level of total radioactivity in the plasma (C_max_ and AUC_0-t_) was similar to the sum of the exposure levels of hyzetimibe and hyzetimibe-M1, indicating that no other major products of metabolism existed in the blood circulation.

### Total Radioactivity Ratio of Whole Blood to Plasma

The total radioactivity ratio of whole blood to plasma was less than 1 and ranged from 0.409 to 0.764. This result suggests that total radioactivity did not bind to blood cells and that the radioactive substances in circulation were mainly distributed in the plasma.

### Urine and Fecal Recovery of Radioactive Dose (Mass Balance)

**FIGURE 2:**
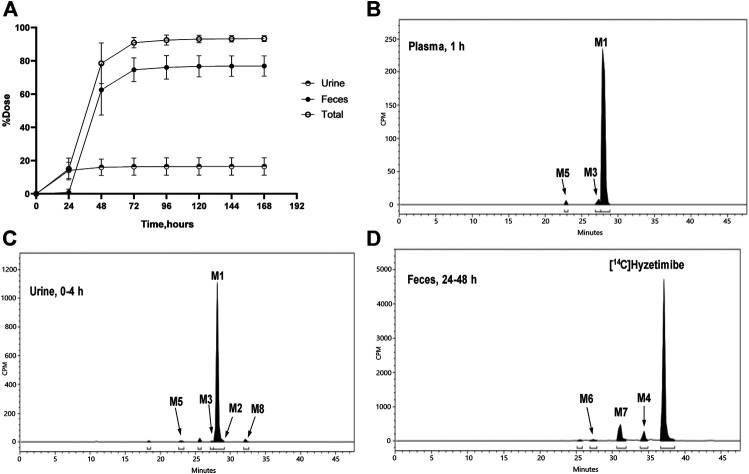
**(A)** Mean (SD) cumulative recoveries of total radioactivity in urine, feces, and combined (n = 6, mean ± SD). Radio-chromatograms of **(B)** 1 h plasma **(C)** 0–4 h urine, and **(D)** 24–48 h feces of six healthy male volunteers. CPM, count per minute.

Recovery of the total cumulative radioactivity in the urine and feces from 0 to 168 h after the dose is presented in Figure 2 and Table 2. The mean cumulative recovery of the total radioactivity in urine and feces from 0 to 168 h period after the dose amounted to 93.29 ± 1.82% of the dose. The mean cumulative recovery of the total radioactivity in feces accounted for 76.90 ± 6.17%; the mean cumulative recovery in the urine accounted for 16.39 ± 5.25%. Excretion of total radioactivity mainly occurred within 72 h; accounted for 90.91 ± 3.18%.

**TABLE 2:** Mean (SD) cumulative excretion of total radioactivity in urine and feces (*n* = 6).

Time (h)	Cumulative excretion of total radioactivity (% dose)		
	Urine	Feces	Total excretion
0–24	14.06 (4.90)	0.87 (1.96)	14.94 (6.53)
0–48	15.92 (4.97)	62.54 (15.19)	78.45 (12.28)
0–72	16.29 (5.16)	74.63 (7.19)	90.91 (3.18)
0–96	16.36 (5.22)	76.05 (7.05)	92.40 (3.07)
0–120	16.39 (5.25)	76.69 (6.35)	93.08 (2.25)
0–144	16.39 (5.25)	76.84 (6.21)	93.23 (1.93)
0–168	16.39 (5.25)	76.90 (6.17)	93.29 (1.82)

SD, standard deviation.

### Metabolite Profiles

As shown in Table 3, eight main metabolites were identified in addition to unchanged drug: glucuronidation of phenolic hydroxyl (M1), glucuronidation of aliphatic hydroxyl (M2), mono-oxidation (M4), glucuronidation of M4 (M3), glucuronidation of phenolic hydroxyl and aliphatic hydroxyl (M5), sulfonation of M4 (M7), oxidation and hydrogenation of M7 (M6), and formylation of M1 (M8). The percentages of unchanged drug and products of metabolism in the total radioactivity exposure in the plasma (% AUC), urine (% dose), and feces (% dose) of healthy male volunteers are summarized in Table 4.

**TABLE 3:** Identification of *in vivo* metabolites of hyzetimibe.

Component	Structure proposal	MW (Da)	Retention time (min)	m/z	m/z^14^C	Typical MS fragment	Matrix
Hyzetimibe	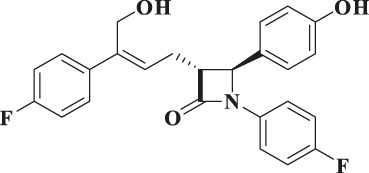	421	37.1	422.1553	422.1605	265–293–404	P, U, F
M1	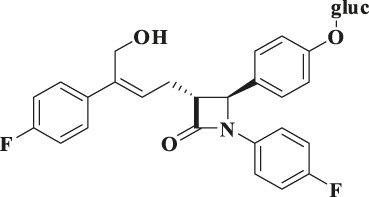	597	27.9–28.1	598.1863	600.1914	422–404–293–265	P, U
M2	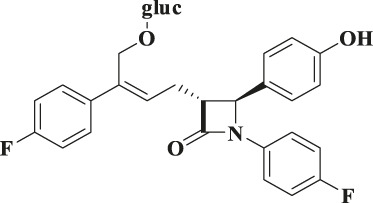	597	28.6–28.9	598.1864	600.1917	422–404	U
M3	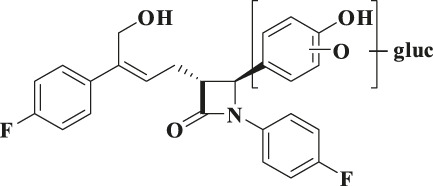	613	27.1–27.4	614.1818	616.3768	438–420–281	P, U
M4	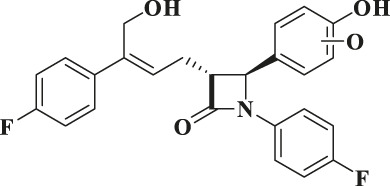	437	34.4	438.1502	440.1551	420–281	F
M5	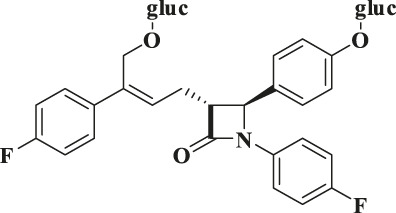	773	22.9–23.1	772.2068	774.2128	596–283	P, U
M6	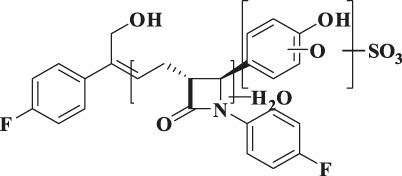	535	27.4	534.1046	536.1125	516–436–299–281	F
M7	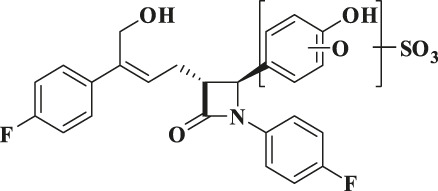	517	31.1	518.1072	520.1150	518–500–420–309–283	F
M8	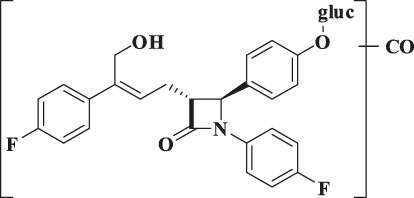	625	32.1–32.4	624.1692	626.1744	596–487–311–283–265	U

F, feces; MS, mass spectrometry; MW, molecular weight; P, plasma; U, urine.

**TABLE 4:** Mean percentages of hyzetimibe and metabolites excreted in plasma, urine, and feces after a single oral dose of 20 mg of ^14^C-hyzetimibe suspension.

Component	Plasma (0–t)^a^	Urine (0–48 h)	Feces (0–120 h)
	% AUC	% Dose	% Dose
Hyzetimibe	+	+	65.0
M1	97.2	15.6	ND
M2	ND	0.19	ND
M3	0.39	0.07	ND
M4	ND	ND	3.78
M5	1.77	0.10	ND
M6	ND	ND	0.17
M7	ND	ND	7.92
M8	ND	0.35	ND

AUC, area under the curve; ND, not detected.

a The plasma samples of 007, 010, and 013 healthy volunteers from 0 to 48 h, 015 and 020 healthy volunteers from 0 to 36 h, and 016 healthy volunteers from 0 to 24 h were mixed according to AUC.

+: only mass spectrometry (MS) data was detected.

The unchanged drug was not detected by radio-chromatography in mixed plasma. The AUC_0-t_ of the main metabolite, M1, in plasma was 931 ± 291 h × ng Eq/ml, accounting for 97.2 ± 1.81% of the total radioactivity exposure in plasma. The total radioactivity exposure in plasma of M3 and M5 was present in minor quantities of 0.39–1.77%. Other metabolites accounted for 0.70% of the total radioactivity exposure in plasma and were not identified. The excretion of total radioactivity in urine from 0 to 168 h was 16.39 ± 5.25% of the dose. The unchanged drug was only detected by mass spectrometer and could not be quantified by radio-chromatography. The main metabolite identified in the urine was M1 (15.6 ± 4.66% of the dose). M2, M3, M5, and M8 represented a small quantity (0.07–0.35% of the dose). Other metabolites accounted for 0.06% of the dose and were not identified. The excretion of total radioactivity in the feces from 0 to 168 h was 76.90 ± 6.17% of the dose. In the feces, the unchanged drug was identified as the main entity (65.0 ± 7.87% of the dose). The main metabolite identified in the feces was M7 (7.92 ± 5.81% of dose). Two other minor metabolites, M4 and M6, represented 3.78 and 0.17% of the dose, respectively. Radio-chromatograms for human plasma, urine, and feces are illustrated in Figure 2.

**FIGURE 3:**
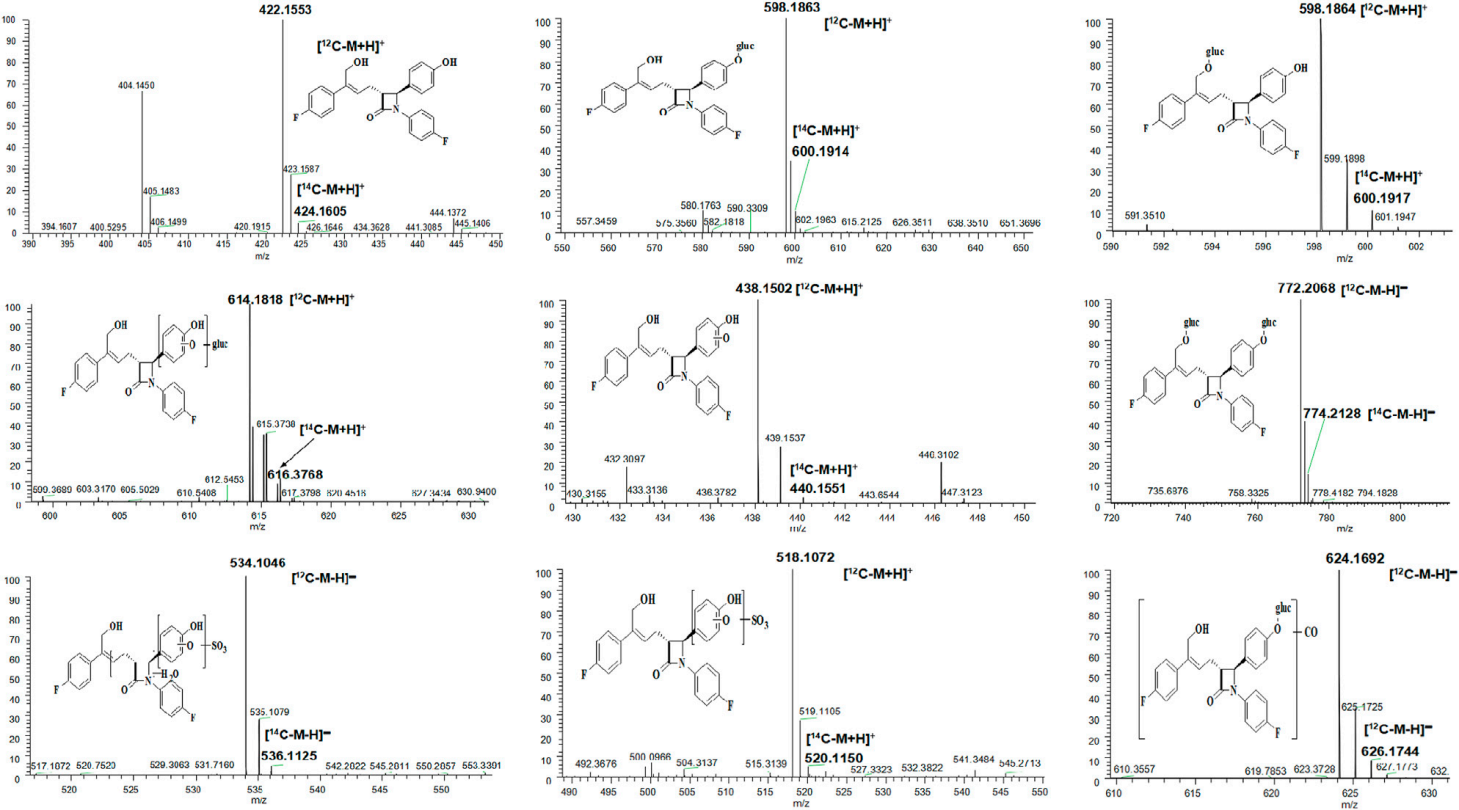
Full-scan chromatogram of unchanged drug and metabolites by high performance liquid chromatography combined with low-energy radioactivity monitor/(±) electrospray ionization-Fourier transform mass spectrometry, HPLC-RAM/(±) ESI-FTMS.

**FIGURE 4:**
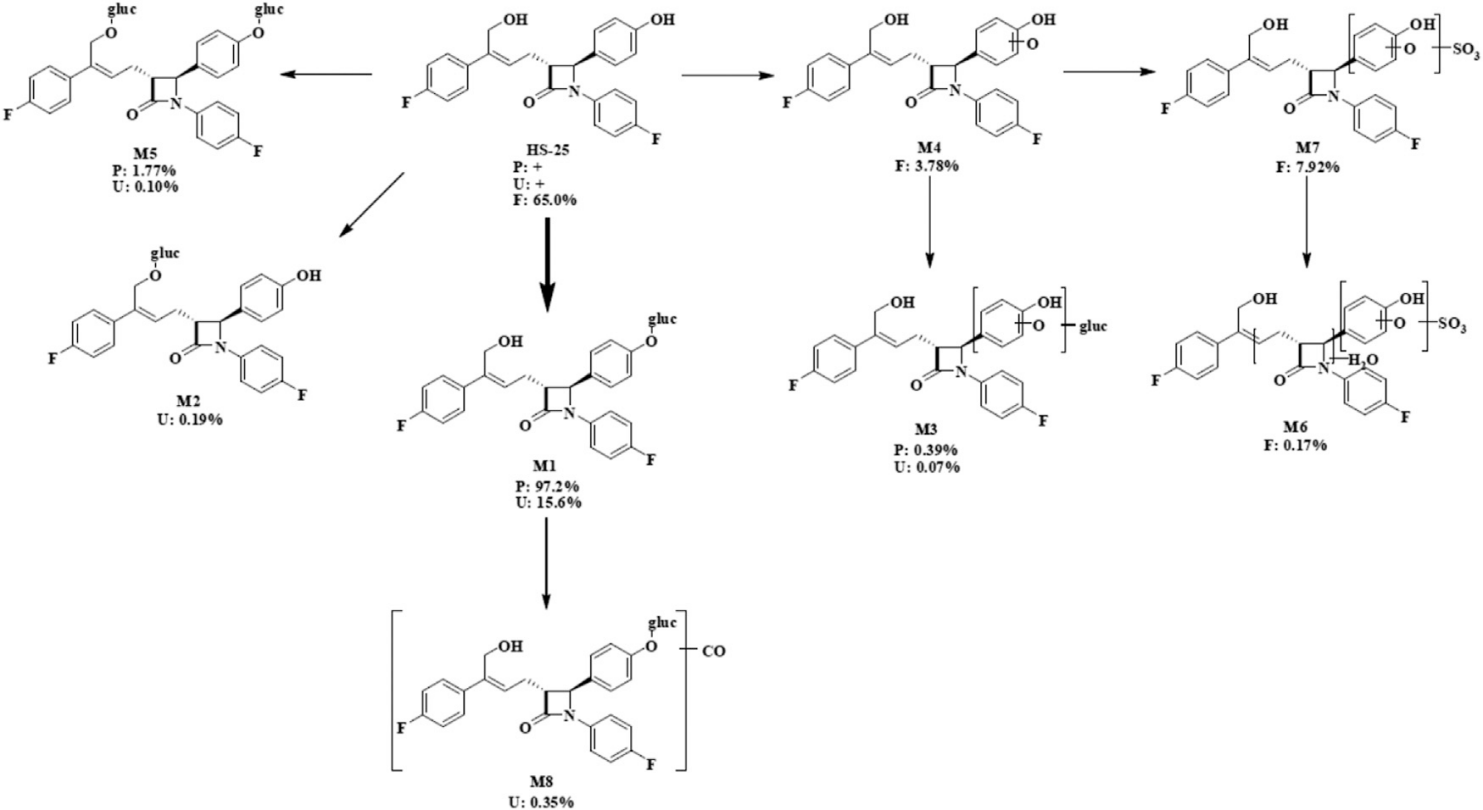
Proposed metabolic scheme. F, feces (% dose); gluc, glucuronic acid (C_6_H_8_O_6_); P: plasma [% area under the curve (AUC)]; U, urine (% dose).

According to the results of metabolite identification, the main metabolic routes for hyzetimibe in healthy male volunteers are as follows: glucuronidation (M1), mono-oxidation (M4), and mono-oxidation with additional sulfonation (M7). The proposed metabolic scheme is shown in Figures 3, 4.

### Safety Evaluation

Hyzetimibe was generally regarded as safe and well tolerated. No deaths, severe AEs, or AEs leading to discontinuation were reported in the study. Three volunteers reported five AEs. All but one (diarrhea) of the events were considered unrelated to the study drug. No abnormal vital sign measurements, ECG results, and physical examination findings were reported. Two volunteers (volunteer 007 and 016) had uric acid at levels that were considered abnormal and of clinical relevance, and one volunteer (volunteer 015) had abnormal, clinically significant high-sensitivity C-reactive protein (hs-CRP) and fecal occult blood test (FOBT) results. These abnormalities were mild and were not considered related to the drug preparation.

## Discussion

To our knowledge, this study was the first to report radiolabeled pharmacokinetic details about hyzetimibe, a cholesterol absorption inhibitor, in humans. Preclinical pharmacokinetic studies had suggested that hyzetimibe was metabolized to its active metabolite by different isoforms of UDP-glucuronosyltransferase in the intestine and liver. The data obtained from this radiolabeled study of the absorption, distribution, metabolism, and excretion of ^14^C-labeled hyzetimibe in healthy male volunteers demonstrated that the main metabolic route of hyzetimibe was glucuronide conjugation (a phase II reaction) in the small intestine and liver. Hyzetimibe and its metabolites are excreted through bile and urine Hyzetimibe was rapidly metabolized into hyzetimibe-glucuronide. The time of maximum concentration (T_max_) for hyzetimibe-M1 occurred from 0.25 to 2.5 h after oral administration. Plasma hyzetimibe-glucuronide accounted for 97.2% of total plasma radioactivity (AUC_0-48_). A multi-peak phenomenon was observed from plasma concentration-time profiles and indicated that the drug had enterohepatic circulation. By the end of sample collection (168 h), the mean cumulative recovery of the total radioactivity in the urine and feces combined was 93.29% of the dose after a single oral administration of 20 mg/∼100 μCi of ^14^C-labeled hyzetimibe suspension in six healthy male volunteers. The mean cumulative recovery of the total radioactivity in urine was 16.39% of the dose, and the mean cumulative recovery in feces was 76.90%. These results indicated that the primary route of elimination of radioactivity was by fecal elimination. The excretion of total radioactivity mainly occurred within 72 h, amounting to 90.91% of the dose. The exposure level of the total radioactivity in plasma (C_max_ and AUC_0-t_) was similar to the sum of the exposure level of hyzetimibe and hyzetimibe-M1, indicating that no other major products of metabolism existed in the blood circulation. The major component in the feces was unchanged hyzetimibe, which accounted for 65% of the dose. The major component in the urine was hyzetimibe-glucuronide, which accounted for 15.6% of the dose.

In contrast, Patrick et al. (2002), Zetia (ezetimibe) tablets: pharmacology review(s) and Zetia (ezetimibe) tablets: prescribing information reported a mean T_max_ of total radioactivity of 2.63 h after oral administration of ^14^C-labeled ezetimibe (20 mg) to healthy male participants. The mean maximum concentration (C_max_) of total radioactivity in that study was 75.1 ng Eq/g, and the mean AUC_0-t_ of total radioactivity in plasma was 780 h × ng Eq/g. The mean T_max_ in the plasma was 9.88 h for ezetimibe and was 2.31 h for ezetimibe glucuronide (SCH 60663). The mean maximum concentration (C_max_) was 5.21 ng/ml for ezetimibe and was 61.2 ng/ml for ezetimibe glucuronide. The mean AUC_0-t_ in plasma was 86.4 h × ng/ml for ezetimibe and was 636 h × ng/ml for ezetimibe glucuronide. The primary metabolic pathway for ezetimibe was identified as glucuronidation of the phenolic hydroxyl, and minimal oxidative metabolism was observed. The cumulative recovery of radioactivity in the feces was approximately 78% of dose, and cumulative recovery in the urine was 11% after a single oral administration of 20 mg of ^14^C-labeled ezetimibe in the healthy male volunteers. The major component recovered in the urine was ezetimibe-glucuronide, accounting for 9% of dose (Patrick et al., 2002).

In our study, the change of hydroxyl group in the drug structure made it easier for hyzetimibe to combine with glucuronic acid, which led to more excretion of hyzetimibe (15.6% of dose) than ezetimibe in the urine. A small amount of other oxidative metabolites of ezetimibe were also identified in urine and feces in the study by Patrick et al. (2002). The pharmacokinetic parameter results for oral ^14^C-radiolabeled hyzetimibe in our study were similar to results with oral hyzetimibe reported by Ruan et al. (2014) with the same dose (Table 5). According to the mean and individual hyzetimibe and hyzetimibe-M1 concentration-time and total radioactivity-time profiles in the plasma, a multi-peak phenomenon appeared for hyzetimibe and hyzetimibe-M1; most of the secondary peaks occurred at 4–6 and 10–12 h. These results indicated that hyzetimibe and hyzetimibe-glucuronide had enterohepatic circulation. Hyzetimibe-glucuronide transformed into hyzetimibe, which was reabsorbed into the blood. The multi-peak times of hyzetimibe, hyzetimibe-glucuronide, and total radioactivity were close to dinner time. Food intake stimulated bile secretion (Fuchs et al., 1998) and then promoted enterohepatic recycling after the transformation of hyzetimibe-glucuronide and hyzetimibe. Because multiple peaks made it was impossible to reliably estimate the terminal-phase rate constant by regression analysis, the shorter mean half-lives of hyzetimibe and hyzetimibe-glucuronide were not consistent with results from a prior clinical study (Ruan et al., 2014), as shown in Table 5. Enterohepatic recycling could affect the efficacy of hyzetimibe, because hyzetimibe acts on the small intestine to lower cholesterol absorption.

**TABLE 5:** Pharmacokinetic parameters of hyzetimibe, hyzetimibe-M1,^14^C-hyzetimibe and ^14^C-hyzetimibe-M1.

Parameter	Unit	Hyzetimibe^a^	^14^C-hyzetimibe	Hyzetimibe-M1^a^	^14^C-hyzetimibe-M1
N	—	10	6	10	6
T_max_	h	1.75 (1.00, 5.00)^b^	3.19 (0.250, 4.95)^b^	2.00 (1.00, 2.50)^b^	1.00 (0.250, 2.50)^b^
C_max_	ng/mL	8.4 (4.4)	2.81 (1.04)	191.0 (72.4)	166 (49.6)
AUC_0-t_	h × ng/mL	106.4 (45.2)	32.9 (11.4)	983.4 (293.9)	950 (303)
AUC_0-∞_	h × ng/mL	116.2 (45.3)	34.3 (11.1)	1,030.4 (302.8)	987 (277)
t_1/2_	h	16.1 (4.3)	9.13 (3.10)	17.4 (4.9)	9.13 (3.56)

NOTE. Data are mean (SD) unless otherwise noted.

AUC_0-t_, area under the concentration-time curve from zero (predose) to the time of the last quantifiable concentration; AUC_0-∞_, area under concentration-time curve from zero (predose) extrapolated to infinity; C_max_, maximum plasma concentration; SD, standard deviation; T_max_, time to maximum plasma concentration.

a Reported by Ruan et al. (2014).

b Median (range).

The total radioactivity ratio of whole blood to plasma was less than one. This result suggests that the total radioactivity did not bind to blood cells.

Eight main metabolites and unchanged drug were identified. The main metabolic routes of hyzetimibe in humans were glucuronidation (M1, 15.6% in urine), mono-oxidation with additional sulfonation (M7, 7.92% in feces), and mono-oxidation (M4, 3.78% in feces). Hyzetimibe was considered generally safe and well tolerated in healthy volunteers.

When the metabolism of hyzetimibe and ezetimibe were compared, the following similarities were noted. 1) The primary metabolic pathway was glucuronidation, and the main metabolite was the glucuronide conjugate. 2) The primary route of elimination of drug-derived radioactivity after oral administration was *via* the feces in the form of unchanged drug. 3) Urine was not the main excretion pathway, and the main metabolite in urine was the glucuronide conjugate of hyzetimibe or ezetimibe. However, some differences existed in their metabolic pathways, such as the following: The amount of elimination of drug-derived radioactivity through the feces and urine differed; the types and contents of secondary metabolites (content <10%) differed; and the metabolic pathways in the human body were not completely consistent.

## Conclusion

This pharmacokinetic study of oral ^14^C-radiolabeled hyzetimibe offers a highly comprehensive profile of the disposition of hyzetimibe and enhances the understanding of hyzetimibe metabolic characteristics. The structural change to the hydroxyl group in hyzetimibe (vs. ezetimibe) improved binding with glucuronic acid and increased urinary excretion compared with ezetimibe. This comparison highlights the need for additional investigation of the pharmacokinetic differences—and the resulting impact on efficacy and safety—between hyzetimibe and ezetimibe.

## Data Availability

The raw data supporting the conclusions of this article will be made available by the authors, without undue reservation, to any qualified researcher.

## References

[B1] Chen J. Lou H. Jiang B. Shao R. Ruan Z. Wang J. a. (2015). Simultaneous determination of hyzetimibe and its main active metabolite in plasma by LC-MS/MS and its application in PK study. Bioanalysis 7, 1857–1867. 10.4155/bio.15.114 26295987

[B2] Code of Federal Regulations (2020). Title 21 - Food and Drugs. Part 361 Sec. 361.1: Radioactive drugs for certain research uses. Available at: https://www.accessdata.fda.gov/scripts/cdrh/cfdocs/cfcfr/CFRSearch.cfm?FR=361.1 (Accessed November 2, 2020).

[B3] Fuchs WS von Nieciecki A Molz KH Popescu G Weil A Barkworth MF (1998). [The effect of bile secretion on the pharmacokinetics of a theophylline sustained-release preparation]. Arzneimittelforschung 48, 597–604. 9676352

[B4] International Commission on Radiological Protection (1991). Radiological protection in biomedical research. ICRP Publication 62. Ann ICRP 22, 1–28. 1845339

[B5] International Commission on Radiological Protection (2007). Recommendations of the international commission on radiological protection. ICRP Publication 103. Ann ICRP 37, 1–332. 10.1016/j.icrp.2007.10.003 18082557

[B6] Patrick J. E. Kosoglou T. Stauber K. L. Alton K. B. Maxwell S. E. Zhu Y. (2002). Disposition of the selective cholesterol absorption inhibitor Ezetimibe in healthy male subjects. Drug Metab. Dispos. 30, 430–437. 10.1124/dmd.30.4.430 11901097

[B7] Ruan Z. Jiang B. Chen J. Zhang X. Lou H. Xiang M. (2014). Pharmacokinetics, pharmacodynamics, safety, and tolerability of hyzetimibe (HS-25) in healthy Chinese subjects. The Journal of Clinical Pharmacology 54, 1144–1152. 10.1002/jcph.310 24752831

[B8] U.S. Food and Drug Administration (2002a). Zetia (ezetimibe) tablets: Clinical Pharmacology Biopharmaceutics Review(s). Available at: https://www.accessdata.fda.gov/drugsatfda_docs/nda/2002/21445_Zetia_biopharmr_P1.pdf (Accessed November 2, 2020).

[B9] U.S. Food and Drug Administration (2002b). Zetia (ezetimibe) tablets: prescribing information. Available at: https://www.accessdata.fda.gov/drugsatfda_docs/label/2002/21445lbl.pdf (Accessed November 2, 2020).

